# Assessing the Role of States in the Age-Friendly Movement: Insights and Recommendations for Policy Development

**DOI:** 10.1093/geroni/igaf122.1110

**Published:** 2025-12-31

**Authors:** Claire Wickersham, Caitlin Coyle

**Affiliations:** LeadingAge LTSS Center at UMass Boston, Boston, Massachusetts, United States; University of Massachusetts Boston, Boston, Massachusetts, United States

## Abstract

States across the U.S. are advancing age-friendly initiatives by joining networks that foster age- and dementia-friendly communities. This presentation examines state-level engagement in the age-friendly movement to inform the “refresh” of the Massachusetts’ plan. A content analysis of existing state plans on aging (including age friendly plans, multisector plan, master plans on aging and state unit on aging plans) was conducted to identify key themes, strategies, and trends in age-friendly advancements across states. States were selected for this review based on having either 1) an AARP Age-Friendly State Designation; or 2) a Multisector Plan on Aging (MPA) in development, implementation, or supported by legislation or an executive order. Based on these criteria, 19 states were included: 12 with AARP Age-Friendly designations, 8 with MPAs in development or implementation, and 4 with MPAs supported by legislation or an executive order. Findings highlight diverse approaches to systems change, including legislative actions (e.g., states passing executive orders or statutory requirements for age-friendly planning), policy changes (e.g., integrating age-friendly principles into housing or transportation policy), programmatic endeavors, initiatives, advocacy efforts, and cross-sector partnerships. The research underscores the role of state governments in driving and sustaining age-friendly progress, offering insights into strategies that have successfully impacted older adults over the past 5-10 years. This presentation emphasizes the importance of shared accountability tools, strengthened cross-sector partnerships, and robust policy frameworks in accelerating and sustaining long-term impact.

